# The Antimicrobial Effect of Gold Quantum Dots and Femtosecond Laser Irradiation on the Growth Kinetics of Common Infectious Eye Pathogens: An In Vitro Study

**DOI:** 10.3390/nano12213757

**Published:** 2022-10-26

**Authors:** Ahmed O. El-Gendy, Yousif Obaid, Esraa Ahmed, Chukuka S. Enwemeka, Mansour Hassan, Tarek Mohamed

**Affiliations:** 1 Laser Institute for Research and Applications LIRA, Beni-Suef University, Beni-Suef 62511, Egypt; 2 Department of Microbiology and Immunology, Faculty of Pharmacy, Beni-Suef University, Beni-Suef 62514, Egypt; 3 Anbar Health Department, Ministry of Health, Ramadi 31001, Iraq; 4 College of Health and Human Services, San Diego State University, San Diego, CA 92182, USA; 5 Faculty of Health Sciences, University of Johannesburg, Doornfontein 2028, South Africa; 6 Department of Ophthalmology, Faculty of Medicine, Beni-Suef University, Beni-Suef 62514, Egypt

**Keywords:** eye infection, laser ablation, antimicrobial treatment, gold nanoparticles, biocompatibility, Gram-positive, Gram-negative, femtosecond laser, quantum dots, MRSA

## Abstract

We studied the antimicrobial effect of gold quantum dots (AuQDs), femtosecond laser irradiation, and the combined effect of laser irradiation and AuQD treatment against common infectious eye pathogens. The INSPIRE HF100 laser system (Spectra Physics) provided a femtosecond laser, which was pumped by a mode-locked femtosecond Ti: sapphire laser MAI TAI HP (Spectra Physics), while a Quanta-Ray nanosecond Nd: YAG laser (Spectra-Physics) was used to precisely synthesize 7.8, 8.7, and 11.6 nm spherical AuQDs. Then, the in vitro growth kinetics and growth rate analysis of *E. coli*, methicillin-resistant *Staphylococcus aureus*, *Enterococcus faecalis*, *Listeria monocytogenes*, and *Candida albicans* (treated with the AuQDs, femtosecond laser irradiation, or combined laser and AuQDs treatment) was measured. The biocompatibility of the AuQDs with the retinal epithelial cell lines (ARPE-19) and their toxicity to the cells was assayed. The results showed that (1) in vitro irradiation using a 159 J/cm^2^ energy density obtained from the 400 nm femtosecond laser suppressed the growth of each of the five pathogens. (2) Similarly, treatment with the AuQDs was antimicrobial against the four bacteria. The AuQDs with an average size of 7.8 nm were more highly antimicrobial and biocompatible and were less cytotoxic than the larger AuQD sizes. (3) The combined femtosecond laser irradiation and AuQD treatment was more highly antimicrobial than each treatment alone. (4) The AuQD treatment did not impair the rate of wound closure in vitro. These findings suggest that combined femtosecond laser irradiation and AuQD treatment is significantly antimicrobial against *Candida albicans*, Gram-positive *L. monocytogenes*, *S. aureus*, and *E. faecalis*, as well as Gram-negative *E. coli*. The nontoxicity and biocompatibility of the AuQD particles tested suggest that this form of treatment may be clinically viable.

## 1. Introduction

The eye is a functionally and anatomically complicated organ. Numerous bacterial, viral, fungal, or parasitic pathogens infiltrate the eye, causing severe ocular infections [1]. Bacterial pathogens (both Gram-positive and Gram-negative) are responsible for nearly 32 to 74% of ocular infections worldwide [2,3]. Bacterial ocular infections include conjunctivitis, keratitis, endophthalmitis, blepharitis, orbital cellulitis, and dacryocystitis [4]. If left untreated, ocular infections can damage the structures of the eye resulting in blindness and visual impairments [5]. The most threatening ocular infections are those affecting the cornea (keratitis) or the eyeball (endophthalmitis) [6]. Bacterial keratitis is the main cause of corneal blindness [5], while endogenous bacterial endophthalmitis (EBE) is an inflammatory reaction in intraocular fluids or tissues, and it is one of the most serious complications of ophthalmic surgery [6,7,8]. Different bacterial pathogens produce different risk levels for ocular infection [9,10]; these infections can be mono- or polymicrobial [5]. Worldwide, the most common Gram-positive bacterial isolates from ocular infections are *Staphylococcus aureus* and *Listeria monocytogenes*, plus a few reported cases of *Enterococcus faecalis* [11], while *Pseudomonas aeruginosa*, *Klebsiella pneumonia*, *Salmonella*, and *Escherichia coli* are the most common Gram-negative bacterial isolates [12]. Ocular fungal infection with *Candida* sp. has also been reported [1].

Accurate diagnosis and appropriate treatment strategies are necessary to manage ocular infections successfully [9,10]. However, more than 70% of bacterial infections are now untreatable [13]. In addition, antibiotic resistance is rapidly emerging among bacterial pathogens as a result of the ongoing overuse and misuse of antibiotics [14], hence the ongoing search for novel alternative antibacterial treatments [15].

Nanomaterial-based treatment strategies have recently received much attention as promising alternative antimicrobial treatment strategies with several advantageous biological characteristics, including high biocompatibility, strong adsorption, and simplicity in surface modification [16]. Nanoparticles (NPs) are small organic, inorganic, or hybrid particles with a diameter of 1–100 nm [17,18]. At such a small scale, NPs exhibit unique physicochemical and biological properties [18,19], such as high surface-to-volume ratios, multivalence, as well as the ability to release high levels of ions at low concentrations [20]. These unique properties influence their biochemical activities [15,21].

In addition, NPs can take on a variety of shapes, such as wires, rods, pyramids, fibrous networks, and spheres with a hollow or solid interior and a rough or smooth surface [15]. Typically, NPs fall into two categories: organic and inorganic [22]. Inorganic metal-based nanoparticles are composed of either pure metals or their compounds (for example, oxides). Their main antimicrobial mechanisms involve the release of reactive oxygen species and the impairment of membrane function [23,24]. The antimicrobial capabilities of various metal/metal oxide NP types, including silver, zinc oxide, titanium oxide, and gold, have been studied [13,25]. Gold is the least reactive metal with a wide range of ligand-binding properties [26,27]. Gold nanoparticles (AuNPs) have been recognized in many biological and medical applications because of their stable chemical characteristics, good biocompatibility, minimum acute cytotoxicity, and spectacular catalytic and plasmonic behavior [22,28,29,30,31]. The antimicrobial efficacy of AuNPs is closely related to their physicochemical properties, such as shape, surface, and size. Tuning these properties can improve its efficacy and biocompatibility while minimizing host toxicity [32].

Nanoparticles with dimensions of 2–10 nm are termed quantum dots (QDs). Due to their very small size, they exhibit many unique photochemical and photophysical properties, such as high quantum yields, excellent photostability, fluorescent emission characteristics, relatively broad excitation wavelengths, and sharp emission peaks [16,30]. Due to their greater curvatures and high surface area, gold quantum dots (AuQDs) have a higher contact surface area with bacteria, thus causing more membrane damage than larger NPs, which increases their antimicrobial efficacy [33,34].

Laser matter interaction can be used to produce contamination-free nanoparticles, alloys, and functionalized nanomaterials for different applications in a simple technique known as laser ablation in liquid (LAL). NPs produced by LAL have remarkable size-tunable properties such as size distribution, dispersion, crystal structure, surface area, porosity, surface charge, shape, and solubility. These properties make LAL an exciting research topic for many applications [35].

Another promising antimicrobial treatment strategy is laser-based antimicrobial photodynamic therapy (lb-aPDT), a noninvasive therapeutic approach for eliminating various pathogens [30]. As a follow-up to our previous studies into the bactericidal efficacy of femtosecond laser-based antibacterial therapy [36,37], here, we present our recent progress in investigating the bactericidal efficacy of femtosecond laser irradiation combined with AuQD-based antimicrobial treatment. Our aim was to (1) evaluate the antimicrobial efficacy of AuQDs prepared at different laser ablation times in liquid compared with femtosecond lb-aPDT, and the combined effect of both treatments and (2) to find the optimal treatment parameters for potential clinical ophthalmic applications.

## 2. Materials and Methods

### 2.1. High-Power Nanosecond Laser System Preparation and Setup for Synthesis of AuQDs

**Figure 1:**
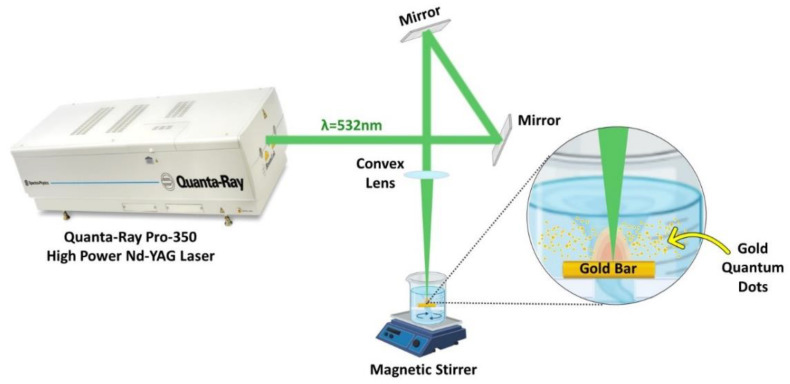
Schematic diagram of the laser ablation in liquid; experimental setup for the synthesis of AuQDs using the 2nd harmonic of the Nd: YAG 532 nm pulsed laser.

A Nd: YAG laser (Quanta-Ray PRO-Series 350-10, Spectra Physics) was used to ablate gold in deionized water at a repetition rate of 10 Hz. A convex lens with a 100 mm focal length was used to concentrate the laser beam onto the Au bulk (>99% purity and has a rectangular shape) to generate AuNPs, as illustrated in the experimental setup, Figure 1. The Au bulk was submerged in a beaker containing 50 mL of deionized water. The beaker was covered with a lid having a hole equal to the laser beam size to protect the convex lens to prevent water spillage. Using a magnetic stirrer, a colloid solution of AuNPs with high solubility was produced. A laser pulse energy of 100 mJ, 532 nm wavelength, and ablation periods of 15, 20, and 30 min were used to produce the AuQDs.

### 2.2. Characterization of Laser Ablated Au Quantum Dots

A transmission electron microscope (TEM) was used to examine the shape and dimension of the AuQDs. TEM images of each preparation were used to measure the size distribution.

### 2.3. Microorganism and Culture Conditions

The microbial pathogens in the present report and their growth conditions are listed in Table 1. Before treatment, the turbidity of the microbial suspension was adjusted to 0.5 McFarland, 1.5 × 10^8^ colony forming units (CFU/mL), before transferring 100 µL fractions into assigned wells of a 96-well microtiter plate.

### 2.4. Femtosecond Laser System Preparation and Setup

The effect of 50 mW average-power 400 nm femtosecond laser irradiation on the growth kinetics of *E. coli*, methicillin-resistant *Staphylococcus aureus*, *Enterococcus faecalis*, *Listeria monocytogenes*, and *Candida albicans* was investigated. Laser pulses were delivered using an INSPIRE HF100 laser system pumped by a mode-locked femtosecond Ti: sapphire MAI TAI HP laser (Spectra-Physics) using ∼1.5–2.9 W average power, 80 MHz repetition rate, and wavelength ranging from 690 to 1040 nm.

**Figure 2:**
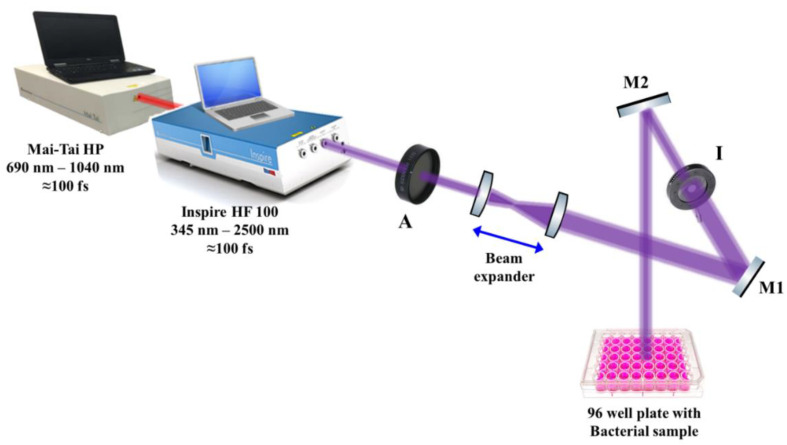
Schematic diagram of the experimental setup for the antimicrobial femtosecond laser treatment. A: attenuator, M1 and M2: highly reflective mirrors, I: Iris.

The laser beam was adjusted approximately 10 cm above each microbial culture in a 96-well microtiter plate, as shown schematically in Figure 2. To ensure the uniform interaction of the laser light with the microbial cultures, a beam expander with two converging lenses was used to expand the initial laser beam of ~2 mm (diameter) to reach 20 mm in diameter, while an iris (I) was used to adjust the laser beam diameter to 6 mm, which is the diameter of each well in the microtiter plate. To guide the laser beam to the samples, highly reflective mirrors, M1 and M2, were used, while a laser beam attenuator (A) was used to control the laser intensity delivered to the samples, as shown in Figure 2. The final laser beam power was measured using a power meter (Newport 843R).

### 2.5. Evaluation of the Growth Kinetics of Gram-Positive and Gram-Negative Bacterial Pathogens after Treatment

The freshly inoculated 96-well plates with microbial cultures were irradiated with horizontally polarized Gaussian laser pulses of a 100 fs pulse duration, at a repetition rate of 80 M Hz and a wavelength of 400 nm with an average power of 50 mW for an exposure time of 15 min.

To gain a deeper understanding of the effect of the AuQDs with or without prior femtosecond laser treatment on the different Gram-positive and Gram-negative bacterial eye pathogens, growth kinetics analysis was performed. A 100 μL fraction of vortexed AuQDs in a double-strength BHI broth was plated in each well. From each prepared bacterial culture, a 100 μL fraction was added to the wells using a multichannel pipette. Positive control wells containing microorganisms without AuQDs and negative control wells containing only BHI broth were also assigned. Using a microplate reader with 620-nm wavelength, the optical density (OD) of each well was measured every 30 min for 16 h while the plates were incubated at 37 °C, as detailed in Table 1.

To explore the effect of AuQD concentration on the growth kinetics of different bacterial pathogens, a concentration of 7.8 nm AuQDs was gradually increased from 17 μg/mL to 33 and 55 μg/mL, and the results were evaluated for those cultures with and without prior femtosecond laser treatment.

Growth curve and growth rate analysis at specific time points (µ_max_) were determined to evaluate the possible discriminant effect of the AuQDs on the pathogens. The following equation was used to obtain µ_max_, where X_t_ is the growth absorbance at a specified time point, X_0_ is the initial growth absorbance, and t is the time at which µ_max_ was obtained.

X_t_ = X_0_ exp (µ_max_·t)

### 2.6. Evaluation of the Growth Kinetics of Candida albicans after Treatment with Laser-Ablated AuQDs and Femtosecond Laser at 400 nm

The growth curve, growth rate, and growth kinetics of *Candida albicans*, the most common fungal eye infection, were also determined after treatment with the AuQDs, with or without prior femtosecond laser treatment.

### 2.7. Cytotoxicity and Biocompatibility of AuQDs to Adult Retinal Cell Line Using MTT Assay

The cellular metabolic activity of the adult retinal pigment epithelial cell lines (ARPE-19) after AuQDs treatment was investigated using an MTT assay to assess the cytotoxicity and biocompatibility of the prepared AuQDs. This colorimetric assay is based on the reduction of a yellow tetrazolium salt (3-(4,5-dimethylthiazol-2-yl)-2,5-diphenyltetrazolium bromide or MTT) to purple formazan crystals by metabolically active cells. In a T-75 tissue culture flask, cells were preincubated with a 20 mL (RPMI) 1640 Medium (Sigma-Aldrich, St. Louis, MO, USA), containing 10% *v*/*v* fetal bovine serum, along with a 1% antibiotic-antimycotic solution (10.000 penicillin units, 10 mg streptomycin, and 25 μg amphotericin B/mL). After 48 h of incubation in a CO_2_ incubator at 37 °C, the medium was discarded, and the cells were detached with a trypsin solution. With a concentration of 4 × 10^4^ cells/cm^2^, the cells were seeded into a 96-well microtiter plate (100 μL/well) and incubated overnight to ensure their adherence to the plate. A two-fold serial dilution approach was used to expose the cell monolayer to the AuQDs and was incubated in a CO_2_ incubator at 37 °C for 24 h. After incubation, a phase-contrast inverted microscope was used to examine the cultures to detect any changes in cell morphology after the AuQD treatment. The cells were then incubated at 37 °C in a medium containing 0.5 mg/mL MTT for 4 h. Then, the excess medium was discarded, and the formed formazan was extracted using DMSO. A microtiter plate reader was used to measure the absorbance at 570 nm. The percentages of cytotoxicity and cell viability were calculated using the following equations.

% cytotoxicity = 1 − (mean absorbance of treated cells/mean absorbance of control).

% viability = 100 − % cytotoxicity.

### 2.8. In Vitro Wound Scratch Assay of Adult Retinal Cell Line after Treatment with Laser-Ablated AuQDs

The laser-ablated AuQDs were tested in an in vitro scratch assay model to investigate if they accelerate or slow the rate of cellular migration. The results of this assay offer physiologic information to improve target treatments and dosing concentrations. Cells in RPMI media were cultured in a 24-well plate with a seeding density of 2 × 10^5^ cells/cm^2^ and incubated in a CO_2_ incubator at 37 °C for 24 h. To horizontally scrape the cell’s monolayer, sterile P200 pipette tips were used. Cell debris was then washed with PBS, and photos of the scratch were taken with a phase-contrast microscope at 40× magnification. The cells were then treated with differently prepared samples of AuQDs at a concentration of 17 μg/mL and compared to the untreated control cells. After an incubation period of 24 h, the second set of images was taken. All the images were processed using “image J” software and analyzed with GraphPad Prism 7 software to determine the treated cells’ migration rate, wound closure percentage, and edge-to-edge analysis.

### 2.9. Antioxidant Activities of AuQDs by DPPH Free Radical Scavenging Assay

DPPH (2,2-diphenyl-1-picryl-hydrazyl-hydrate) free radical assay was used to determine the percentage of free radical scavenging activity for each prepared AuQD sample, according to the standard method of Boly et al. [38], where DPPH could be reduced in the presence of an antioxidant molecule that is capable of donating hydrogen, changing its color from deep violet to light yellow. At room temperature, a fraction of 100 µL freshly prepared DPPH reagent (0.1% in methanol) was added to 100 µL of each AuQD sample in a 96-well-plate (n = 6) and incubated for 30 min in the dark. Then, the change in DPPH color intensity was recorded at 540 nm using a microplate reader: FluoStar Omega. Data are represented as means ± SD using the following equation.

$$\%\;Inhibition=\frac{Average\;absorbance\;of\;blank-Average\;absorbance\;of\;the\;test\;sample}{Average\;absorbance\;of\;blank}\times 100$$

### 2.10. Data Analysis

A growth curve was constructed to monitor the growth and proliferation of each microbe over time, followed by growth rate and growth kinetics analyses. The data are presented as mean ± standard error. One-way analysis of variance (ANOVA) testing followed by Tukey’s testing were used for multigroup comparisons of the means using the GraphPad Prism 7 software. *p* < 0.05 was considered significant. Each experimental procedure was carried out in triplicate within a sterile laminar flow hood (class II biological safety cabinet, MSC-Advantage TM).

## 3. Results and Discussion

### 3.1. Growth Kinetics of Femtosecond Laser-Treated Pathogens

**Figure 3:**
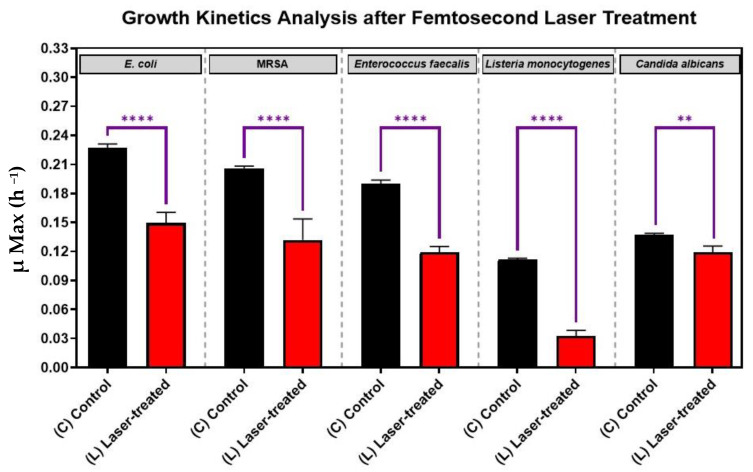
Growth analysis of four different bacterial pathogens: *E. coli*, *Staphylococcus aureus*, *Enterococcus faecalis*, *Listeria monocytogenes*, and the fungus, *Candida albicans*, after femtosecond laser treatment with a 400 nm wavelength and an average power of 50 mW for 15 min. Bar graph comparing the growth rate during the log phase of the control culture (C) with that of the bacterial cultures exposed to laser treatment (L). Significance was tested by ANOVA followed by Tukey testing (**** *p* < 0.0001, ** *p* < 0.01).

As is shown in Figure 3, for each pathogen (*X*-axis), the laser-treated cultures grew significantly slower and had significant reductions in growth kinetics (*Y*-axis) compared to the control cultures (*p*-value < 0.0001). This finding shows that each of the five eye pathogens, *Escherichia coli*, *Staphylococcus. aureus*, *Enterococcus faecalis*, *Listeria monocytogenes*, and *Candida albicans* were susceptibly suppressed by laser irradiation in vitro. The antimicrobial effect of the treatment was more significant for each of the four bacteria than for the fungus *Candida albicans* (Figure 3).

In one of our recent studies [39], we examined the antibacterial effect of lb-aPDT on the growth kinetics of *S. aureus* as different femtosecond laser parameters were used to optimize the treatment. We showed that 15 min of exposure to 390 nm or 400 nm femtosecond laser at an average power of 50 mW was sufficient to cause the maximum reduction in bacterial viability. In the present study, we exposed different microbial cultures to a 159 J/cm^2^ energy density using a 400 nm femtosecond laser, which is a wavelength in the visible region of the electromagnetic spectrum and is relatively safer than shorter wavelengths for therapeutic applications [40,41]. Thus, this finding suggests that the safer 400 nm wavelength may be a viable alternative for clinical use.

It is worth mentioning that, throughout the present study, the maximum power density used was 0.063 W/cm^2^ (average power of 50 mW, at 10 mm spot size), which is less than the maximum permissible exposure limits for tissues, including those within the eye, according to the International Commission on Non-Ionizing Radiation Protection guidelines and far away from exerting any harmful effect [42], suggesting that the laser treatment parameters of this study will not cause additional damage to the eyes and will not affect the eyesight.

### 3.2. Synthesis and Characterization of AuQDs for Antimicrobial Testing

**Figure 4:**
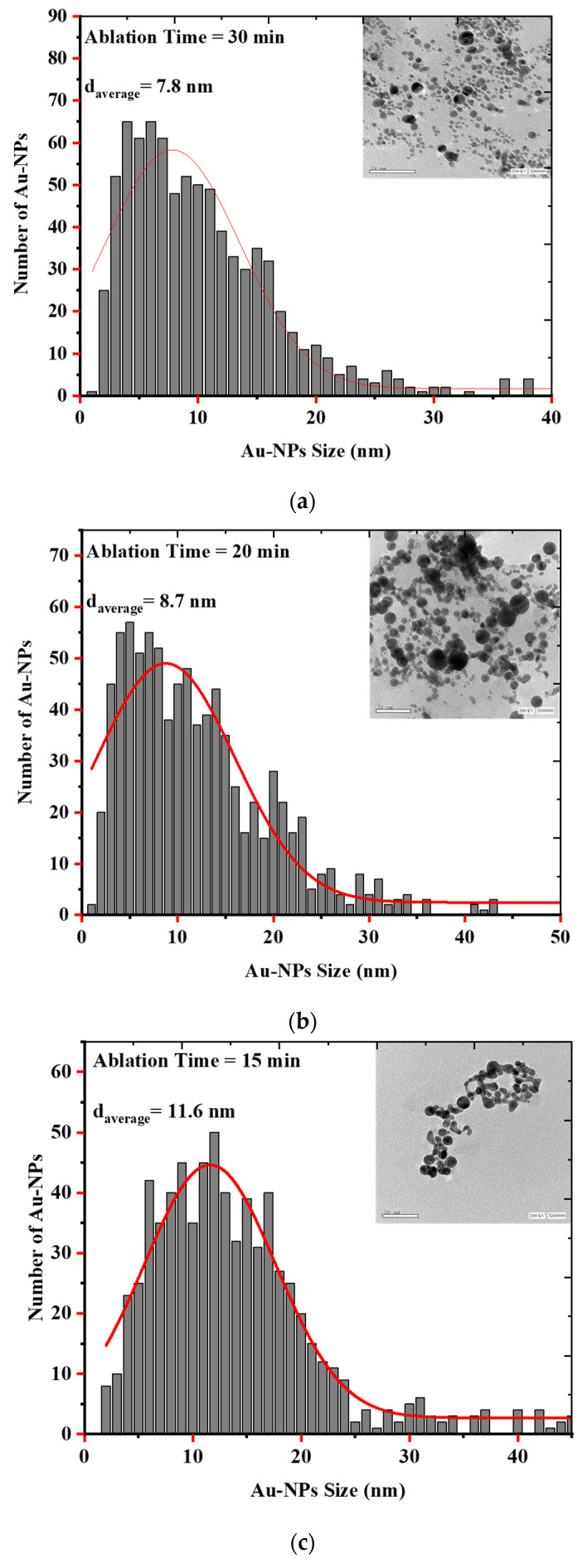
EM images and histograms of the AuQD size distribution produced by laser ablation in distilled water (LAL- DW) using a 100 mJ laser pulse energy and different ablation times of (**a**) 30 min, (**b**) 20 min, and (**c**) 15 min.

Laser Ablation in Liquid (LAL) is a unique and effective technique to produce, excite, fragment, and conjugate a wide range of nanoparticles in a clean, scalable way. Parameters such as irradiance, pulse duration, liquid type, sample geometry, and focusing conditions affect the synthesis of the particles [35]. As evidenced by the electron microscopic images and the histograms showing the size distribution of the AuQDs prepared with this method, the AuQDs were spherical, and the average NP sizes were 7.8, 8.7, and 11.6 nm for 30, 20, and 15 min ablation times, respectively (Figure 4). The Gaussian fit of each histogram shows that the particles were in the quantum dot range.

The high surface-to-volume ratios and multivalence of nanoparticles promote interactions with biomolecules within bacteria. This gives NP several advantages as an antimicrobial, including (1) a broad-spectrum antibacterial efficacy against both Gram-positive and Gram-negative bacteria, (2) the potential to combat recalcitrant infections and biofilms, and (3) the ability to overcome bacterial resistance [15,43,44].

### 3.3. Effect of Nanoparticle Size on the Growth Kinetics of the Microbial Pathogens

**Figure 5:**
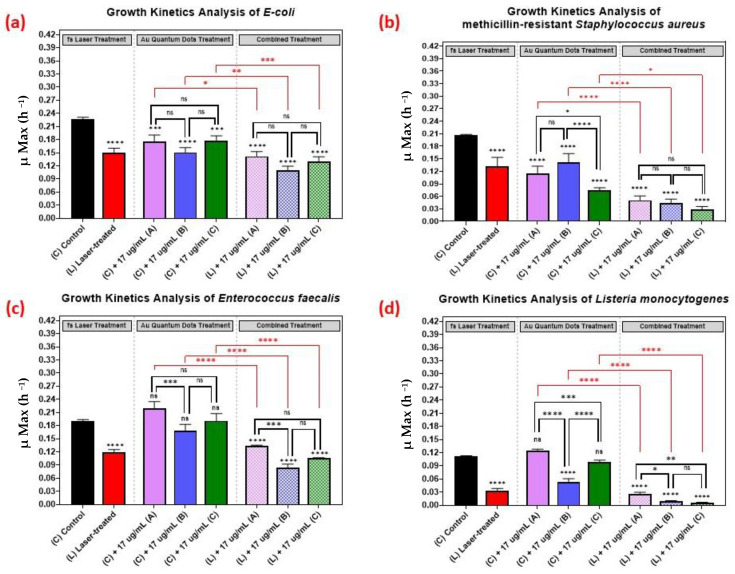
Bar graph of the four different bacterial pathogens: (**a**) *E. coli*, (**b**) *Staphylococcus aureus*, (**c**) *Enterococcus faecalis*, and (**d**) *Listeria monocytogenes*, comparing growth rate during the log phase of the control culture (C) with that of the bacterial cultures exposed to different treatments: laser treatment (L), AuQDs treatment (C + A, B, or C), and laser + AuQDs treatment (L + A, B, or C). All four pathogens were exposed to femtosecond laser treatment using a wavelength of 400 nm and average power of 50 mW for 15 min. The cultures were exposed to three samples of AuQDs: sample (A) (7.8 nm average size), sample (B) (8.7 nm average size) and sample (C) (11.6 nm average size) at the same concentration of 17 µg/mL. Significance was tested by ANOVA followed by Tukey testing (**** *p* < 0.0001, *** *p* < 0.001, ** *p* < 0.01, * *p* < 0.05, ns; no significance).

As shown in Figure 5, we comparatively evaluated the effect of the different sizes of the AuQDs for sample (A) (7.8 nm average size), (B) (8.7 nm average size), and sample (C) (11.6 nm average size). The concentration was kept constant at 17 µg/mL, and the growth kinetics analyses of the different bacterial pathogens were measured. Even though the average sizes of the AuQDs were relatively close, there were significant differences in their antibacterial effect. In general, the antibacterial effect was higher in samples (B) and (C), especially with *Staphylococcus aureus* and *Listeria monocytogenes* (Figure 5). This could be attributed to the homogeneity and aggregability of the samples, as shown in the TEM images in Figure 5. The antibacterial activity of gold nanoparticles is partly due to the release of gold ions [45]. When NPs are in contact with bacterial cells, they release Au^+^ ions that are evenly distributed around the bacteria and then penetrate the cell walls and enter the cells leading to cell death [13,46]. Smaller NPs release Au^+^ ions faster [13]. The release of the Au^+^ ions would be even higher at higher concentrations, and this could enhance the antibacterial effect [47]. It is noteworthy that, in general, the combined treatment with the AuQDs and the laser was consistently greater in its antimicrobial effect than no treatment or treatment with either the laser or the AuQDs alone.

### 3.4. Effect of Nanoparticle Concentration on the Growth Kinetics of the Microbial Pathogens

**Figure 6:**
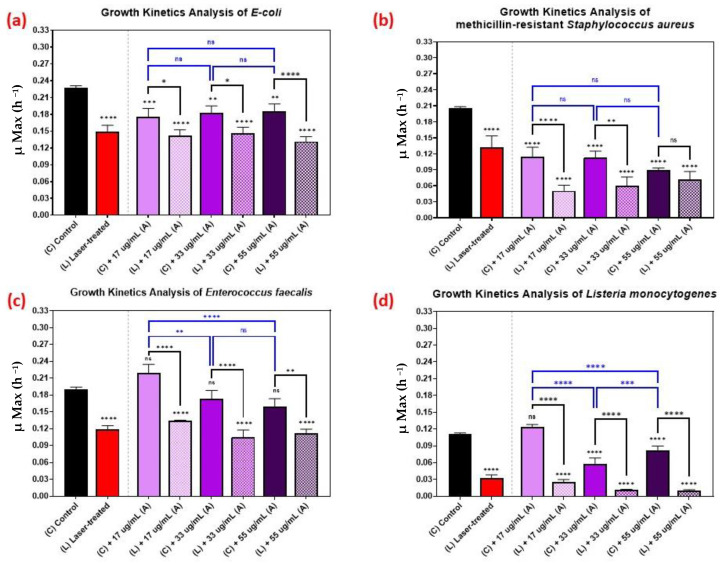
Bar graph of the four different bacterial pathogens: (**a**) *E. coli*, (**b**) *Staphylococcus aureus*, (**c**) *Enterococcus faecalis*, and (**d**) *Listeria monocytogenes*, comparing growth rate during the log phase of the control culture (C) with that of the bacterial cultures exposed to different treatments: laser treatment (L), AuQDs treatment (C + A), and laser + AuQDs treatment (L + A). All four pathogens were exposed to femtosecond laser treatment with a wavelength of 400 nm and average power of 50 mW for 15 min. The cultures were exposed to AuQDs-treated sample (A) (7.8 nm average size) with concentrations of 55, 33, and 17 µg/mL. Significance was tested using ANOVA followed by Tukey testing (**** *p* < 0.0001, *** *p* < 0.001, ** *p* < 0.01, * *p* < 0.05, ns; no significance).

Figure 6 shows the kinetic growth analyses of those pathogens treated with various concentrations of AuQDs: 55, 33, and 17 µg/mL. For *Listeria monocytogenes*, the effect of increasing the concentration was vivid, but not so with the other bacteria (Figure 6). This observation highlights the fact that it is not always optimal to increase concentration. In some cases, a lower AuQD concentration may be sufficiently antimicrobial, depending on the bacterium. Moreover, increasing the concentration in such cases may compromise safety and biocompatibility. This finding suggests a need to pay attention to the physicochemical properties of the applied AuQDs as well as the treatment strategy.

### 3.5. Growth Kinetics Analysis of Candida albicans

**Figure 7:**
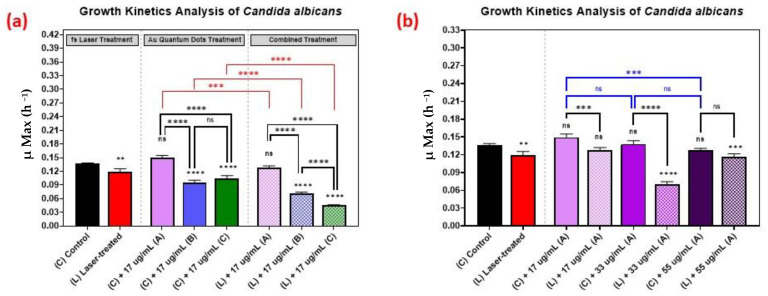
Growth analysis of *Candida albicans*, comparing the control culture (N) to the laser-treated (L), AuQDs-treated (N + A, B, or C), and laser + AuQDs-treated (L + A, B, or C) cultures. Sample (A) (7.8 nm average size), sample (B) (8.7 nm average size), and (C) (11.6 nm average size). (**a**) Bar graph comparing the growth rate during the log phase of the control culture (N) with that of the bacterial cultures exposed to different treatments, where the AuQD treatment was carried out using three samples: sample (A), sample (B) and sample (C) at the same concentration of 17 µg/mL. (**b**) Bar graph comparing the growth rate during the log phase of the control culture (N) with that of the bacterial cultures exposed to different treatments, where the AuQD treatment was carried out using sample (A) with concentrations of 55, 33, and 17 µg/mL. Significance was tested by ANOVA followed by Tukey testing (**** *p* < 0.0001, *** *p* < 0.001, ** *p* < 0.01, ns; no significance).

The growth kinetics of *Candida albicans* followed a similar trend as the growth kinetics of the bacteria, as is shown in Figure 7, with 33 µg/mL concentration yielding better results than the 17 µg/mL or 55 µg/mL concentrations.

The effect of the combined treatment of laser irradiation followed by AuQD treatment is shown in Figure 5, Figure 6 and Figure 7, showing a significant reduction in the growth kinetics (*Y*-axis) of cultures exposed to the different treatments (*X*-axis) when compared to the control cultures for each microbial pathogen. In all cases, pair-wise comparisons of each AuQD treatment with each corresponding combined AuQD and laser treatment were highly significant: *p* < 0.0001, especially with *Staphylococcus aureus* and *Listeria monocytogenes*. However, the pair-wise comparisons between the growth kinetics of the cultures exposed to the three AuQD treatments after laser exposure were not significant in most cases (Figure 5); this shows that the antibacterial efficacy of the AuQDs can be maximized when combined with prior femtosecond laser treatment (with the appropriate parameters). This observation is of practical significance since the enhanced efficacy of the combined treatment suggests that a lower concentration of AuQDs combined with laser irradiation may be more effective than laser or AuQD treatment alone.

### 3.6. Cytotoxicity, Biocompatibility, and Antioxidant Ability of AuQDs

**Figure 8:**
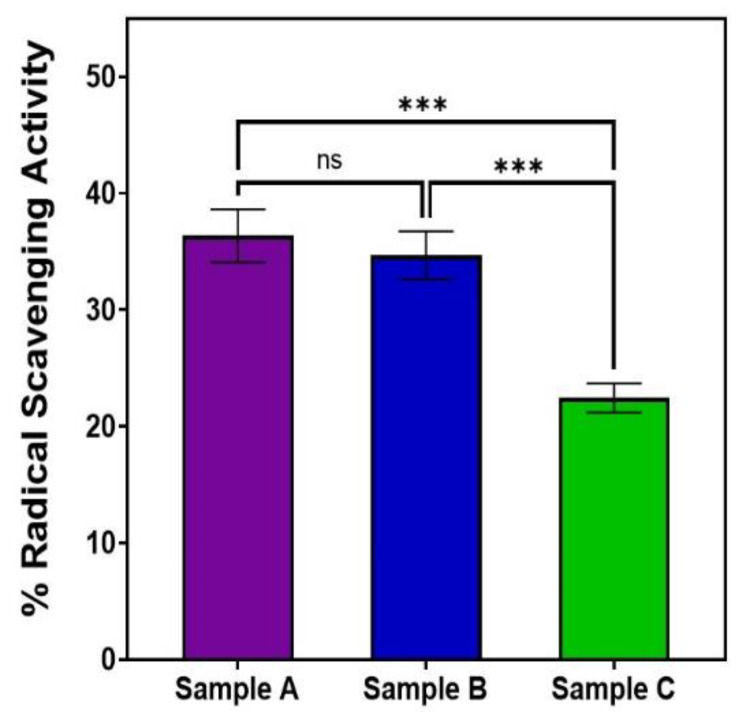
Bar graph comparing the free radical scavenging activity of the three samples of AuQDs prepared at different ablation times: sample A (7.8 nm), sample B (8.7 nm), and sample C (11.6 nm). Significance was tested by ANOVA followed by Tukey testing (*** *p* < 0.001, ns; no significance).

Our MTT and DPPH assays showed that AuQD sample (A), with an average size of 7.8 nm, was less cytotoxic and more biocompatible than the other two samples: sample (B) (average size of 8.7 nm) and sample (C) (average size of 11.6 nm), Table 2. With an average size of 7.8 nm, sample A reduced retinal epithelial cell survival to 13.2% when compared to 16.5% for sample B and 24.2% for sample C. This finding suggests that AuQDs with an average size of 7.8 nm would be more tolerable in practical situations than AuQDs with larger average sizes. Moreover, as is shown in Figure 8, the same 7.8 nm size of the AuQDs has more antioxidant activity: 36.35%, when compared to the other two samples; the antioxidant effect of sample (B), with an average size of 8.7 nm, was 34.67%, and for sample (C), with an average size of 11.6 nm, it was 22.45%. Since oxidative stress is a hazardous condition for eukaryotic cells, the antioxidant capacity of AuQDs is a good feature. It enables the neutralization of excess ROS and free radicals, which is essential for healthy functioning in many biosystems. NPs, including AuQDs, could be a novel approach to limit such oxidative stress.

### 3.7. Effect of AuQDs on Wound Closure

**Figure 9:**
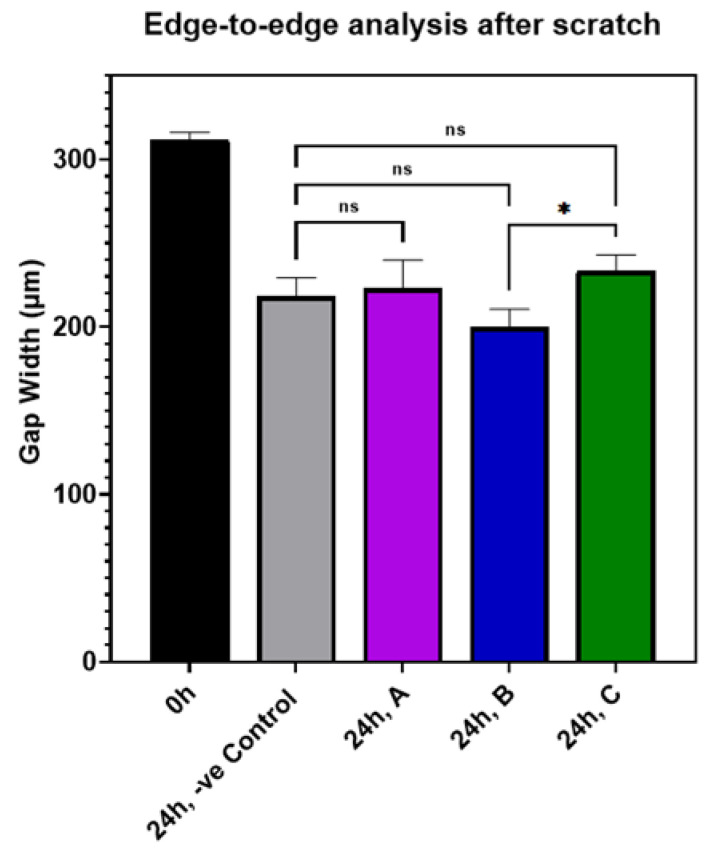
Bar graph comparing the edge-to-edge analysis in an in vitro wound scratch assay after 24 h of incubation of ARPE-19 cells exposed to AuQD samples prepared at different ablation times: sample A (7.8 nm), sample B (8.7 nm), and sample C (11.6 nm), compared to the control cells. Significance was tested by ANOVA followed by Tukey testing (* *p* < 0.05, ns; no significance).

The effect of AuQDs on wound closure in an experimental in vitro scratch wound model is shown in Table S1 and Figure S1. The results indicate that there was no significant difference between the AuQD-treated samples and the control samples, suggesting that AuQDs do not impair clinical wound healing. The migration rates and wound closure percentages of the different AuQD samples are shown in Table S1, and Figure 9 shows an edge-to-edge analysis of the wound closure rates of the AuQD treatments after 24 h. There was no difference in wound closure rate between the samples.

Overall, our results show that, firstly, in vitro irradiation using a 159 J/cm^2^ energy density from a 400 nm femtosecond laser suppressed the growth of *E. coli*, *S. aureus*, *E. faecalis*, *L. monocytogenes*, and *C. albicans*. The four bacteria were more susceptible to treatment than the fungus *C. albicans*. The antimicrobial effect of lasers and other light sources is often attributed to the production of reactive oxygen species (ROS); this is believed to be the major mechanism underlying the antimicrobial effect of certain wavelengths of light. Reactive oxygen species (ROS) are the byproducts of cellular oxidative metabolic activities. At an appropriate level, ROS have a positive effect on cells [13]. However, when cells are exposed to external stimuli, the level of ROS increases significantly [13]; excess ROS can negatively affect cell differentiation, signaling, and survival [48].

ROS is produced by two simultaneously-occurring photochemical processes [49], either (1) electron transfer (type I), which produces oxygen, peroxide, or hydroxide radicals, or (2) energy transfer reactions (type II), which generates singlet oxygen (^1^O_2_) [50]. These processes rely on the photochemical interaction of laser radiation with chromophores which trigger the conversion of light energy to chemical energy [51,52]. The effectiveness of any laser-based treatment in triggering the production of ROS is affected by irradiation parameters, such as power, intensity, fluence, exposure duration, mode of operation (continuous wave or pulsed), and, most importantly, wavelength [53,54]. Our finding suggests that the treatment parameters used in this study were suitable to deactivate the pathogens in vitro, a finding which provides a baseline for potential in vivo studies.

A second major finding of this study is that treatment with quantum dot gold particles at specified concentrations is antimicrobial against the four bacteria. The antimicrobial effect is more pronounced when the average size of the AuQDs is 7.8 nm compared to larger sizes, such as 8.7 nm and 11.6 nm.

NPs exhibit their antibacterial effects through predominantly physical or biochemical processes after being in contact with bacteria cells either by electrostatic attraction, van der Waals forces, receptor–ligand, or hydrophobic interactions [15,55,56]. The antimicrobial activity of NPs can be attributed to three simultaneously occurring mechanisms [55]: (1) cell wall and membrane disruption [57], (2) oxidative stress induction [58], and (3) damage to intracellular components [59]. The bacterial cell membrane acts as a physical barrier to antimicrobials [56]. Numerous nanomaterial-based strategies focus on disrupting this negatively charged barrier, i.e., bacterial cell membrane [60,61]. The electrostatic adsorption of positively charged gold nanoparticles on the membrane can cause membrane damage, leading to cytoplasmic leakage and loss of cell integrity [13,62]. Therefore, therapeutic strategies targeting the bacterial cell membrane are promising for long-term usage as antimicrobials with little or no risk of potential bacterial resistance [48].

Furthermore, AuNPs that are in contact with bacteria directly generates ROS (from their surface) by releasing ions [15]. The ROS produced triggers a cascade of bactericidal effects through mechanisms that result in the peroxidation of membrane lipids, the destruction of membrane proteins [13], and the deactivation of membrane receptors [63]. The presence of ROS within cells can cause protein aggregation and DNA destruction [13], hindering cell division and proliferation and ultimate cell death [13,46]. AuNPs could cross the different structures of bacterial cells, reaching the cytosol, where the inclusion bodies of AuNPs were noticed [64]. The antibacterial action of AuNPs could be achieved in two stages. Firstly, they slow down metabolism by altering membrane potential and lowering adenosine triphosphate (ATP) synthase activities. Secondly, they prevent the tRNA binding to ribosomes during the protein translation steps in bacteria [65]. In addition, proteins containing sulfur and DNA-containing phosphorus may react with AuNPs. To induce oxidative stress, the AuNPs might bind to the thiol groups of enzymes, such as NADH-dehydrogenases, leading to the disruption of their respiratory chains and subsequent release of oxygen species. This extensive cellular damage would ultimately result in cell death [66].

The survival and function of bacteria depend on intracellular signaling networks and cellular homeostasis [15]. When nanomaterials interact with the intracellular components within bacterial cells, such as ribosomes, lysosomes, enzymes, proteins, and/or DNA, their functions can be disrupted, resulting in electrolyte imbalance, enzyme inhibition, protein deactivation, and changes in gene expression [55]. These multiple antimicrobial mechanisms of NPs would require multiple gene mutations in the same bacterial cell to develop resistance [55].

The fact that Gram-negative *E coli* and Gram-positive bacteria, such as *S. aureus*, *L. monocytogenes*, and *E faecalis*, are both susceptible to growth suppression when treated with AuQDs, shows the high potency of AuQDs as an antimicrobial. Bacteria are unicellular prokaryotic organisms that can be classified as either Gram-positive or Gram-negative, depending on the structure of their cell walls [31]. While Gram-negative bacteria only have a thin layer of peptidoglycan, and a thin cytoderm (10 nm), Gram-positive bacteria contain a significant amount of peptidoglycan and a thick cytoderm (20–80 nm) [13]. Therefore, in general, the thin-walled Gram-negative bacteria are more susceptible to invasion by the metal ions released by the NPs [67]. As previously noted, the antibacterial activity of gold nanoparticles is partly due to the release of gold ions [45]. Our finding suggests that the Au+ can penetrate both Gram-positive and Gram-negative bacteria, regardless of their shapes, whether they are round or coccal, e.g., *E coli*, *S. aureus*, and *E faecalis* or rodlike (bacillus), as is the case with *L. monocytogenes*.

Thirdly, we showed that the antimicrobial effect of the treatment is greater when the pathogens are exposed to both femtosecond laser irradiation followed by AuQD treatment at various concentrations (Figure 5, Figure 6 and Figure 7). This finding suggests the potential enhanced antimicrobial effect of both treatments.

With the ongoing advancement in pulsed laser system technology, the numerous benefits of pulsed laser systems (particularly femtosecond lasers) have been noted in biological and therapeutic applications, opening a new era for the clinical application of laser treatment devices. The duration of exposure and heat dissipation play major roles in thermal injury processes. Femtosecond laser pulses have the unique ability to deposit energy into a microscopic volume on a very short time scale within a single laser pulse without affecting the surrounding tissue. Both the pulse width and the repetition rate determine the amount of heat accumulated in the biological specimen. If the heat duration is short, thermal energy can diffuse more rapidly; if the duration between two pulses is long enough for the heat produced by the previous pulse to decay, there will be no accumulation of heat [68,69,70].

Our fourth finding is that AuQDs with an average size of 7.8 nm were less cytotoxic and more biocompatible than the larger sizes. For any clinical antibacterial application, AuQDs must have certain features that enable the highest biocompatibility and biodistribution, as well as the lowest cytotoxicity [71,72]. These features are related to their physicochemical characteristics, such as shape, surface charge, and, most importantly, average size. The distribution of NPs within organs is related to their average size as well as their final concentration and clearance in blood circulation [73]. NPs have to be small enough to evade rapid splenic filtration yet sufficiently large enough to escape renal clearance; this is why NPs with an average size of 6–200 nm are more suitable for clinical applications [74,75]. Along with this finding, we showed that the rate of wound closure was the same in the AuQD-treated samples and controls, again indicating that AuQD treatment could be safely used in vivo. Since we used in vitro and cell culture models, in vivo studies will be needed in subsequent works to advance the clinical applicability of our findings.

Nanotechnology presents new opportunities and the potential to transform both the detection and treatment of a wide range of infectious diseases [76]. Despite its great potential, several challenges must be overcome for any AuQD-based antimicrobial clinical application to attain clinical standards. These include the nonspecific interactions between most NPs and cell membranes that result in the passive accumulation of AuQDs [77,78], the non-biodegradability of most NPs [22], and the long-term systematic safety of nanoparticles within the body. Further studies are needed to explore the possible clinical applications of AuQDs against specific microbial communities in tissues, such as microbial biofilms and the microbial expression of various virulence factors. The precise control of the size, shape, and surface properties of NPs provides a wide range of design options for broader and safer antimicrobial efficacy. With the intensive study of LAL and the contamination-free synthesis technique, the tuning and tailoring of these physicochemical properties for certain applications could be attained. Nonetheless, to the best of our knowledge, this is the first study to evaluate the antibacterial, antioxidant, cytotoxicity, and wound-healing capabilities of AuQDs of different sizes prepared by the same contamination-free method of LAL.

## 4. Conclusions

This study opened a new perspective on the synthesis of AuQDs using a laser ablation technique and using the AuQDs safely to treat different ocular infections. Our findings show that (1) in vitro irradiation using a 159 J/cm^2^ energy density from a 400 nm femtosecond laser suppressed the growth of Gram-negative *E. coli*, Gram-positive *S. aureus*, *E. faecalis*, and *L. monocytogenes*, and the fungus *C. albicans*. (2) Treatment with quantum dot gold particles is antimicrobial against the four bacteria. The antimicrobial effect is more pronounced when the average size of the AuQDs is 7.8 nm compared to larger sizes, such as 8.7 nm and 11.6 nm. (3) The antimicrobial effect of the treatment is greater when the femtosecond laser irradiation is combined with AuQD treatment, indicating that antimicrobial action is enhanced by this. (4) AuQD particles, with an average size of 7.8 nm, are more biocompatible and less cytotoxic than larger particles. (5) AuQDs do not impair the rate of wound closure in vitro. These findings suggest that the combined treatment of femtosecond laser irradiation and AuQDs could be an enhanced, noninvasive, antimicrobial therapeutic approach that is clinically viable and worthy of further in vivo studies.

## Figures and Tables

**Table 1:** Microorganisms and their culture conditions.

Microorganism	Strain	Culture Conditions	
		Culture Media	Temperature (°C)
*Staphylococcus aureus* MRSA	ATCC 43300	BHI broth	37
*Listeria monocytogenes*	ATCC 7644	BHI broth	30
*Enterococcus faecalis*	V583	BHI broth	37
*E. coli*	ATCC 6933	BHI broth	37
*Candida albicans*	ATCC 60913	BHI broth	30

**Table 2:** Cytotoxicity and biocompatibility of AuQDs to the adult retinal cell line using MTT assay. Data are expressed as mean ± SD values of the three independent experiments.

	Negative Control	DMSO	Sample A	Sample B	Sample C
**Survival % of retinal epithelial cells at 20 µg/mL** **(Mean ± SD)**	100 ± 0.64	99 ± 1.20	86.8 ± 1.30	83.5 ± 2.90	75.8 ± 1.87

## Data Availability

Not applicable.

## Supplementary Materials

The following supporting information can be downloaded at: https://www.mdpi.com/article/10.3390/nano12213757/s1, Figure S1: Differences of the gap width in an in vitro wound scratch assay at 0 and 12 h using ARPE-19 cells after exposure to AuQDs samples prepared at different ablation time (a) 30 min. sample A of 7.8 nm size, (b) 20 min. sample B of 8.7 nm size and (c) 15 min. sample C of 11.6 nm size compared to control cells (d) without any treatment. Images were capture using the phase contrast of an inverted optical microscope, at magnification of 10×; Table S1: The migration rate and wound closure percentage of adult retinal cell line after treatment with different AuQDs.
