# Supplementary material for: The Antimicrobial Effect of Gold Quantum Dots and Femtosecond Laser Irradiation on the Growth Kinetics of Common Infectious Eye Pathogens: An In Vitro Study
Source: Nanomaterials (Basel). 2022 Oct 26;12(21):3757. doi: 10.3390/nano12213757 (PMC9654226; doi:10.3390/nano12213757)
Supplement: Supplementary file 1 [file nanomaterials-12-03757-s001.zip › nanomaterials-1961804-supplementary.pdf]

Supplementary Information

The Antimicrobial Effect of Gold Quantum Dots and Femtosecond Laser Irradiation on the Growth Kinetics of Common Infectious Eye Pathogens: An In Vitro Study

Ahmed O. El-Gendy [1,2,*], Yousif Obaid [3], Esraa Ahmed [1], Chukuka S. Enwemeka [4,5], Mansour Hassan [6] and Tarek Mohamed [1]

[1] Laser Institute for Research and Applications LIRA, Beni-Suef University, Beni-Suef 62511, Egypt
[2] Department of Microbiology and Immunology, Faculty of Pharmacy, Beni-Suef University, Beni-Suef 62514, Egypt
[3] Anbar Health Department, Ministry of Health, Ramadi 31001, Iraq
[4] College of Health and Human Services, San Diego State University, San Diego, CA 92182, USA
[5] Faculty of Health Sciences, University of Johannesburg, Doornfontein 2028, South Africa
[6] Department of Ophthalmology, Faculty of Medicine, Beni-Suef University, Beni-Suef 62514, Egypt
* Correspondence: ahmed.elgendy@pharm.bsu.edu.eg; Tel.: +20-1223476015

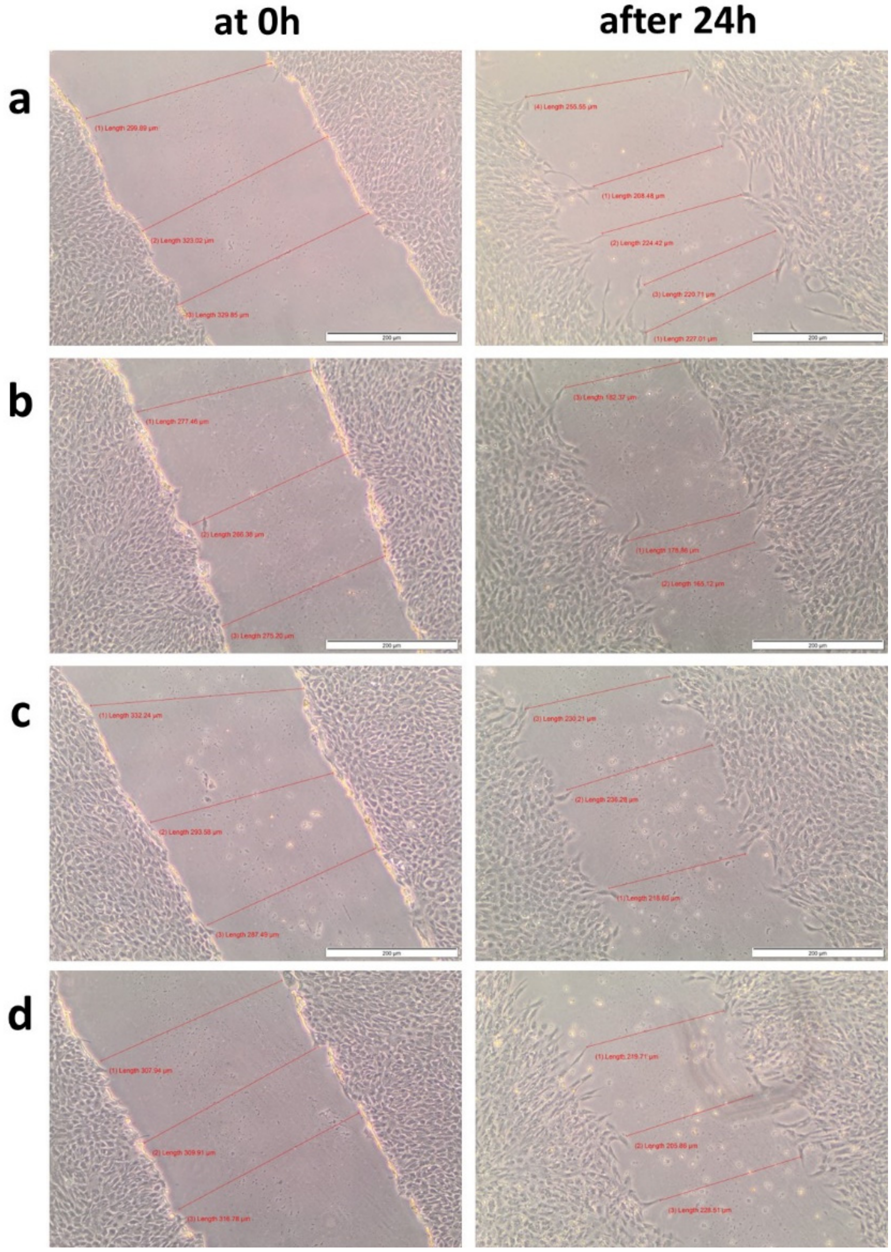

Figure S1. Differences of the gap width in an in vitro wound scratch assay at 0 and 12 h using ARPE-19 cells after exposure to AuQDs samples prepared at different ablation time (a) 30 min. sample A of 7.8 nm size, (b) 20 min. sample B of 8.7 nm size and (c) 15 min. sample C of 11.6 nm size compared to control cells (d) without any treatment. Images were capture using the phase contrast of an inverted optical microscope, at magnification of 10×.

Table S1. The migration rate and wound closure percentage of adult retinal cell line after treatment with different AuQDs.

	**Negative Control**	**Sample A**	**Sample B**	**Sample C**
$\boldsymbol{Migration\ Rate\ (R_m) = \frac{W_i - W_f}{t}}$	3.896	3.765	4.066	3.17
$\boldsymbol{Wound\ Closure\ \% = \left[\frac{A_{t=0h} - A_{t=\Delta h}}{A_{t=0h}}\right] \times 100\%}$	16.929	12.313	16.089	14.979
